# Characteristic Expressions of GABA Receptors and GABA Producing/Transporting Molecules in Rat Kidney

**DOI:** 10.1371/journal.pone.0105835

**Published:** 2014-09-04

**Authors:** Kozue Takano, Midori Sasaki Yatabe, Asami Abe, Yu Suzuki, Hironobu Sanada, Tsuyoshi Watanabe, Junko Kimura, Junichi Yatabe

**Affiliations:** 1 Department of Pharmacology, Fukushima Medical University School of Medicine, Fukushima, Japan; 2 Department of Nephrology, Hypertension, Diabetology, Endocrinology and Metabolism, Fukushima Medical University School of Medicine, Fukushima, Japan; 3 Division of Health Science Research, Fukushima Welfare Federation of Agricultural Cooperatives, Fukushima, Japan; Center for Molecular Biotechnology, Italy

## Abstract

Gamma-aminobutyric acid (GABA) is an important neurotransmitter, but recent reports have revealed the expression of GABAergic components in peripheral, non-neural tissues. GABA administration induces natriuresis and lowers blood pressure, suggesting renal GABA targets. However, systematic evaluation of renal GABAergic components has not been reported. In this study, kidney cortices of Wistar-Kyoto rats (WKY) were used to assay for messenger RNAs of GABA-related molecules using RT-PCR. In WKY kidney cortex, GABA_A_ receptor subunits, α1, β3, δ, ε and π, in addition to both types of GABA_B_ receptors, R1 and R2, and GABA_C_ receptor ρ1 and ρ2 subunit mRNAs were detected. Kidney cortex also expressed mRNAs of glutamate decarboxylase (GAD) 65, GAD67, 4-aminobutyrate aminotransferase and GABA transporter, GAT2. Western blot and/or immunohistochemistry were performed for those molecules detected by RT-PCR. By immunofluorescent observation, co-staining of α1, β3, and π subunits was observed mainly on the apical side of cortical tubules, and immunoblot of kidney protein precipitated with π subunit antibody revealed α1 and β3 subunit co-assembly. This is the first report of GABA_A_ receptor π subunit in the kidney. In summary, unique set of GABA receptor subunits and subtypes were found in rat kidney cortex. As GABA producing enzymes, transporters and degrading enzyme were also detected, a possible existence of local renal GABAergic system with an autocrine/paracrine mechanism is suggested.

## Introduction

Gamma-aminobutyric acid (GABA) acts as a major inhibitory neurotransmitter in the central nervous system. GABA is synthesized in the brain from glutamic acid via glutamic acid decarboxylase (GAD). There are two types of GAD with different molecular weights, GAD67 and GAD65. Released GABA acts on two general classes of its receptors, GABA_A_/GABA_C_ and GABA_B_. GABA_A_ and GABA_C_ receptors are ligand-gated chloride channels composed of a combination of various subunits as pentamers. The unique sensitivity of GABA_A_ receptor to its agonist such as benzodiazepines is known to depend on the combination of subunits including α, β, γ, δ, ε, π, and θ [1], [2]. Meanwhile, GABA_B_ is a metabotropic G-protein coupled receptor coupling with Gi/o. GABA_B_ receptor controls neuronal activity via activation of K^+^ channels, inhibition of Ca^2+^ channels, or both. Similar to the GABA_A_ receptor-mediated increase in the conductance of Cl^−^, the activation of K^+^ channels from the GABA_B_ signaling induces hyperpolarization of the cell membrane, which produces chronic stabilization of neuronal activity [3]. GABA secreted from neurons subsequently undergoes re-uptake via GABA transporters (GAT) and is metabolized by the GABA degrading enzyme, 4-aminobutyrate aminotransferase (ABAT) [4].

Considerable evidence suggests that GABA also mediates diverse responses in non-neuronal tissues. GABA_A_ receptor subunits have been identified in tissues such as the lung [5], retina [6], uterus [7], [8], spermatozoa [9], adrenal medulla [10], pituitary lobes [11], and pancreatic β-cells [12]. Mizuta et al. [13] detected functional GABA_B_ receptors in airway epithelium and airway epithelial cell lines.

The kidney is a key organ responsible for blood pressure regulation by controlling water and sodium balance. It is previously reported that GABA causes natriuresis and lowers blood pressure in human as well as rodents [14], [15]. In addition, GABA_A_ receptor agonist muscimol and GABA_B_ agonist baclofen increased fractional excretion of water and sodium in isolated rat kidney perfusion [16]. These suggest that GABA may play a pivotal role in the renal pathophysiology of hypertension. To date, there are some reports of the presence of GABAergic system in the kidney. GABA immunoreactivity was found in the renal medulla and cortex but not in neuronal structures [17], and depolarizing stimuli such as ouabain increased GABA release from rat kidney slice [18]. Sarang et al. examined the expression of GABA_A_ receptor α1, α2, α4, α5, β1, γ1, γ2 and δ subunits in the kidney cortex from Sprague-Dawley rats and found the expressions of α1, α5, β1, γ1 and γ2 subunits. They also showed that GABA and a GABA_A_ receptor agonist, muscimol, increased Cl^−^ uptake in primary cultured renal proximal tubular cells [19]. The authors also reported the expression of β2 and β3 subunits in the renal proximal tubule [20], but some of the GABA_A_ receptor subunits such as α6, γ3, ε, π and θ have not been examined.

GABA may have an important role in the kidney, but a systematic evaluation of GABA-related molecules has not been performed. Therefore, in this report, the expressions of GABA-related molecules, GABA producing enzyme, receptor components, transporters and degrading enzyme, were examined in rat kidney.

## Materials and Methods

### Animals

All animal experimental procedures were approved by the Fukushima Medical University School of Medicine Animal Committee. Seven-week old Wistar-Kyoto rats (WKY) and spontaneously hypertensive rats (SHR) were obtained from Japan SLC Inc., Sendai, Japan and used for the experiments at 8 weeks of age. Each animal was housed under controlled temperature (21°C) and light conditions (light 7∶00–19∶00, dark 19∶00–7∶00) with either 0.3% or 8% NaCl chow (Oriental Yeast Co., Tokyo, Japan) for a week with tap water ad libitum. Rats were anesthetized using pentobarbital (50 µg/kg) intraperitoneal injection. After opening the abdominal cavity and drawing blood from the abdominal aorta, kidneys and brain were harvested and stored for further analyses. The animals were euthanized by pentobarbital overdose (intraperitoneal administration of over 200 mg/kg pentobarbital sodium). Eight rats per group were used for the experiment.

### Total RNA extraction and reverse transcription

Total RNA was extracted from the rat kidney cortex and brain preserved in RNAlater (Life Technologies, Carlsbad, CA, USA) using RNeasy plus mini kit (QIAGEN K.K., Tokyo, Japan) following the manufacturer’s instructions. Kidneys from 5 rats were used in the study. Universal RNA from rat normal tissues (BioChain, San Francisco, CA, USA) and brain RNA were used as positive controls. RNA concentration was measured using UV spectrometer, and 0.25 µg of total RNA was reverse transcribed in 20 µl reaction volume using iScript cDNA synthesis kit (Bio-Rad, Hercules, CA, USA). The reverse transcriptase step (5 min at 25.0°C, 30 min at 42.0°C, 5 min at 85.0°C) was carried out using My Cycler (Bio-Rad).

### Human RNA samples

Human Kidney Total RNA and Human Brain Total RNA were purchased from Clontech Laboratories, Inc. (Mountain View, CA, USA). The Human Kidney Total RNA was extracted from a normal kidney of 40-year old Caucasian female with a sudden death. The Human Brain Total RNA was extracted from a normal whole brain from a 43-year-old Caucasian male with a sudden death.

### Qualitative RT-PCR

Qualitative PCR was performed using the renal cortical mRNA isolated from WKY fed normal chow. Rat GABA_A_ receptor α1-6, β1-3, γ1-3, δ, ε, π, θ subunits, GAD65, GAD67, GAT1-3, and ABAT nucleotide sequences were identified in Gen Bank database. PCR primers were designed using Primer Blast (Table 1). One µl of the reverse transcriptase products was amplified by PCR using Go Taq Green Maser Mix (Promega, Fitchburg, WI, USA) and 60 nM each of forward and reverse primers. The PCR condition was 2 min at 50.0°C. 10 min at 95.0°C, then 35 cycles of 15 s at 95.0°C and 1 min at 60.0°C, followed by 5 min at 72.0°C. Amplifications for rat GABA_B_ R1 and R2 subtypes and GABA_C_ ρ1-3 subunits were carried out using TaqMan Gene Expression Assay (Life Technologies) following the manufacturer’s protocol (Table 1). All PCR reactions were conducted in iCycler (Bio-Rad). Five µl of PCR products were analyzed by electrophoresis in 10% TBE Gel (Life Technologies) stained with ethidium bromide, and visualized on a UV transilluminator. Rat brain RNA or commercially available reference RNA were used as positive control.

**Table 1:** Primer information.

Gene	Forward primer (3′–5′)	Reverse primer (3′–5′)	Accession No.or assay ID	Ampliconsize (bp)
GABA_A_ α1	tgacagtcattctctcccaagtc	tcagaacggtcgtcactcc	NM_183326	87
GABA_A_ α2	gactgtcattctctcccaagtgt	tcattgtcaaaacagttgttactcc	NM_001135779	92
GABA_A_ α3	cctacttgccatgtatcatgactg	ggtcatggtgagaacagtggt	NM_017069	113
GABA_A_ α4	cttctggatcaataaggagtctgtt	tcatcgtgaggactgtggtt	NM_080587	68
GABA_A_ α5	gccttggaagcagctaaaatc	gtacccacagcattcccagt	NM_017295	146
GABA_A_ α6	agtcccagaaagccgaaag	ttcttcagatggtacttggagtca	NM_021841	124
GABA_A_ β1	ccctctggatgagcaaaact	ccctctcctccattccagta	NM_012956	90
GABA_A_ β2	catcgatatggtttctgaagtcaa	gggattacattgtaggacagtctctt	NM_012957	96
GABA_A_ β3	atcgagctcccacagttctc	tcaatgagagtcgagggtagg	NM_017065	91
GABA_A_ γ1	gatgcgcactggataacaac	tggagttgaaggtagcattctg	NM_080586	110
GABA_A_ γ2	acagaaaatgacgctgtgga	atctgacttttggctagtgaagc	NM_183327	70
GABA_A_ γ3	agcgagtggagaccaagc	ctcttccaccctcctggac	NM_024370	124
GABA_A_ δ	aatgacattggggactacgtg	gccacattcactggaggac	NM_017289	125
GABA_A_ ε	ccgctgagatgttgcctaa	tccagtttaatgtgaggtccaa	NM_023091	88
GABA_A_ π	gttgcagctggaaagttgg	gagtccacgtaccgaatcgt	NM_031029	82
GABA_A_ θ	tgatgtccgactgagaccaa	tctgagatctgttcaatactggagac	NM_031733	87
GAD65	gtacgccatgctcattgccc	agagaggatcaaaagccccg	NM_012563	299
GAD67	gctggaaggcatggaaggtttta	acgggtgcaatttcatatgtgaacata	NM_017007	222
GAT1	ttcaagggtgtgggcctcgc	ccacggcagagtcgtggtga	NM_024371	120
GAT2	gcagcgaacacaagcgcatcc	atcccacctgcagccgctact	NM_133623	126
GAT3	tcgtgttgagcgtagcgggag	ggaatgcccctccgccgtt	NM_024372	90
ABAT	cctgggcatcctgcctccaga	ttgggcgccaccgacatca	NM_031003	70
GABA_B_ R1			Rn00578911_m1	113
GABA_B_ R2			Rn00582550_m1	87
GABA_C_ ρ1			Rn00568768_m1	61
GABA_C_ ρ2			Rn00568783_m1	86
GABA_C_ ρ3			Rn00594210_m1	88

Abbreviations: GABA; γ-aminobutyric acid, GAD; glutamate decarboxylase, GAT; GABA transporter, ABAT; 4-aminobutyrate aminotransferase.

### Quantitative PCR

Quantitative PCR was performed to compare mRNA expression levels in GABA_A_ receptor subunits among WKY and SHR fed normal- or high-salt chow. Quantitative PCR was also performed using human kidney and brain RNA commercially available from Clontech Inc. (Mountain View, CA). One µl of reverse-transcription sample was used for real-time quantitative PCR using the iQ5 Real-Time PCR Detection System and iQ SYBR Green Supermix (Bio Rad) or StepOnePlus Real-Time PCR System and TaqMan Fast Advanced Master Mix (Life Technologies). PCR reactions were performed in triplicate, and mRNA was quantified based on the Ct value, normalized to GAPDH, and expressed as relative amounts.

### Immunoprecipitation

Protein lysate was incubated with Protein G-protein Mag Sepharose (GE Health care Life Sciences, Uppsala, Sweden) closslinked to anti-GABA_A_ receptor π subunit antibody for 12 hours at 4°C. The immunoprecipitated protein samples eluted from the pellets were suspended in sample buffer and resolved by SDS-PAGE. Western blotting was performed with anti-GABA_A_ receptor α1 or β2/3 subunit antibody.

### Membrane protein preparation

WKY kidney membrane protein was separated using Mem-PER Eukaryotic Membrane Protein Extraction Kit (Thermo Scientific, Carlsbad, CA) according to the instruction from manufacturer. The hydrophobic fraction containing membrane proteins was diluted to decrease the detergent concentration. The membrane proteins in SDS sample buffer were resolved by SDS-PAGE and detected by immunoblot.

### Western Blotting

The rat kidney cortex and brain were immediately snap-frozen in liquid nitrogen and stored at −80°C until use. The tissue was homogenized in lysis buffer (50 mM Tris-Cl pH 8.0, 150 mM NaCl. 0.02% sodium azide and 1% NP-40) containing cOmplete protease inhibitor mix (Roche, Tokyo, Japan) and centrifuged at 14000 g for 10 min at 4°C. Protein concentrations were determined by BCA protein assay (Thermo Fisher Scientific, Inc., Waltham, MA, USA). Five to 30 µg/lane of protein were separated on NuPAGE Novex 4–12% Bis-Tris Gels (Life Technologies) and transferred onto PVDF membrane (MILLIPORE, Billerica, MA, USA). The membranes were blocked with Immunoblock (DS Pharma Biomedical Co., Ltd., Osaka, Japan). The membranes were probed with a 1∶1000 dilution of anti-GABA_A_ receptor α1 subunit rabbit polyclonal antibody (MILLIPORE), a 1∶500 dilution of β2/3 subunit mouse monoclonal antibody (MILLIPORE), a 1∶200 dilution of π subunit rabbit polyclonal antibody (Santa Cruz Biotechnology, Inc, Santa Cruz, CA, sc-25708), ρ1 antibody (Santa Cruz, sc-25707), ρ2 antibody (Santa Cruz, sc-21343) or 1∶2000 dilution of ABAT antibody (Sigma-aldrich, St. Louis, MO, USA. Immuno Shot reagents (COSMO BIO Co., LTD, Tokyo, Japan) were used to enhance the immune reaction in some assays. The membranes were washed and probed with 1∶5000 goat anti-rabbit or anti-mouse horseradish peroxidase-conjugated IgG (Santa Cruz, Santa Cruz, Ca, USA). Proteins were detected by the ECL system (GE healthcare, Buckinghamshire, UK).

### Immunohistochemistry

Three-µm tissue sections were incubated with primary antibodies including 1∶50 to 1∶100 dilution of GABA_A_ α1, β2/3 and π subunits, GABA_B_ receptor R1 subtype (Cell-Signaling), R2 subtype (Cell Signaling), GAD67 (Santa Cruz, GAD65 (MILLIPORE) GAT2 (MILLIPORE) or ABAT. Immuno Shot reagents (COSMO BIO) were used to enhance the immune reaction in some assays. Chromogenic immunostaining was performed with an avidin–biotin immunoperoxidase kit (Vectastain Elite kit or ABC/Peroxidase kit, Vector Laboratories, Burlingame, CA, USA) and diaminobenzidine (Sigma Fast DAB Tablets, Sigma-Aldrich). The kidneys were lightly counterstained with hematoxylin. Fluorescent immunostaining was performed using secondary antibody of Alexa Flour 568 conjugated goat anti-rabbit antibody and Alexa Flour 488 conjugated goat anti-mouse antibody (Life Technologies). GABA_A_ π subunit antibody (Abcam: ab26055) was used for immunofluorescence studies. Normal rabbit IgG (Santa Cruz: sc-2027) and mouse monoclonal IgG1 (Abcam: ab81032) were used as negative control. Nuclei were stained by DAPI (Life Technologies). FluoView FV1000 confocal microscope (Olympus, Tokyo, Japan) was used for examination and image acquisition.

## Results

### Expressions of GABA_A_ receptor α1, β3, δ, ε and π subunits were detected in rat kidney cortex

**Figure 1:**
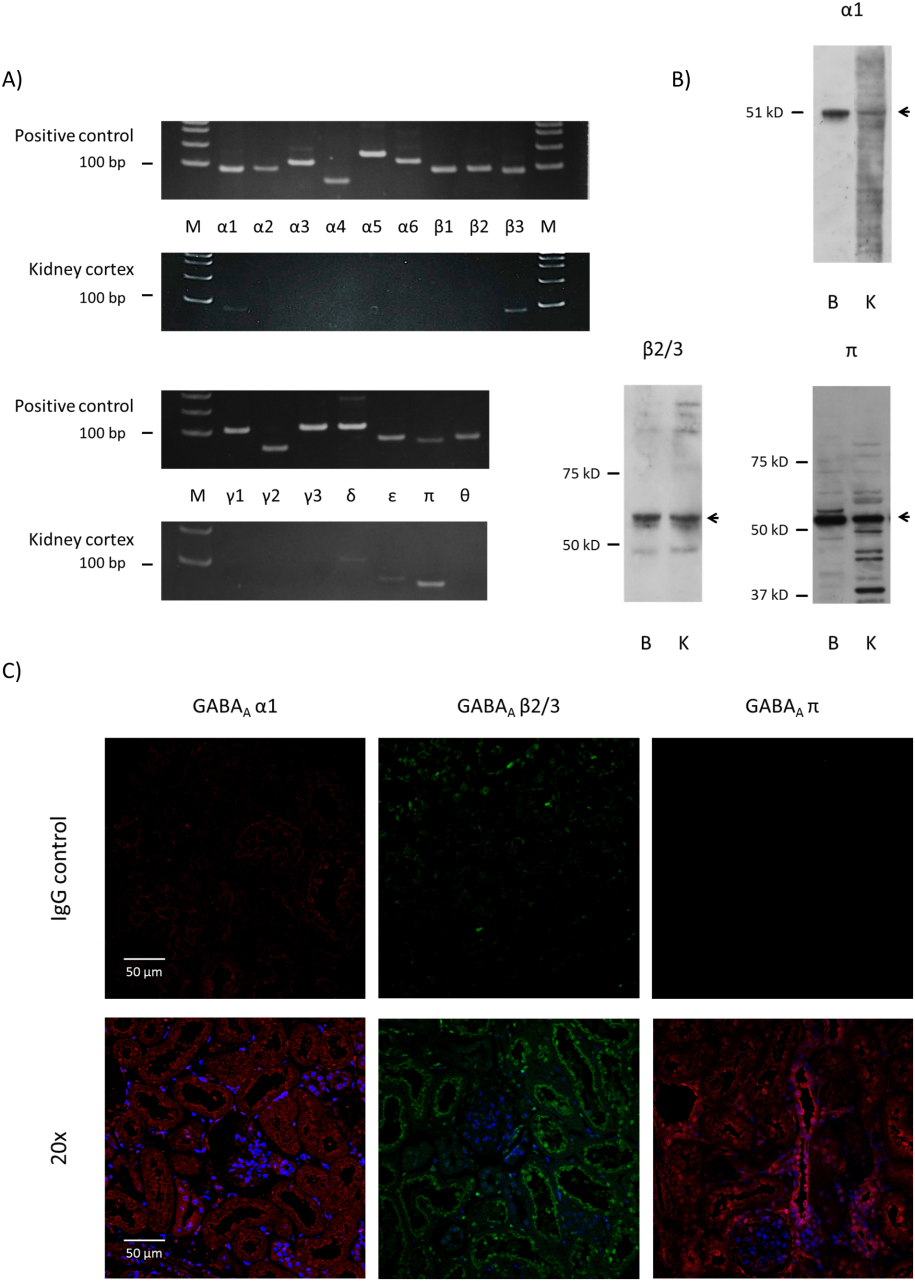
GABA_A_ receptor subunits are expressed in rat kidney. A) RT-PCR results for GABA_A_ receptor subunits in rat (WKY on normal diet) kidney cortex. Appropriately-sized bands were detected for GABA_A_ receptor α1, β3 and π subunits in at least five independent experiments. Rat brain RNA or commercial universal RNA was used as positive control. M: molecular marker. B) Immunoblot for GABA_A_ receptor α1, β3 and π subunits in rat kidney cortex. B: brain control, K: kidney cortex. C) Immunofluorescent examination of GABA_A_ receptor α1, β3 and π subunits in rat kidney cortex. Staining with normal IgG instead of primary antibody served as negative control.

**Figure 2:**
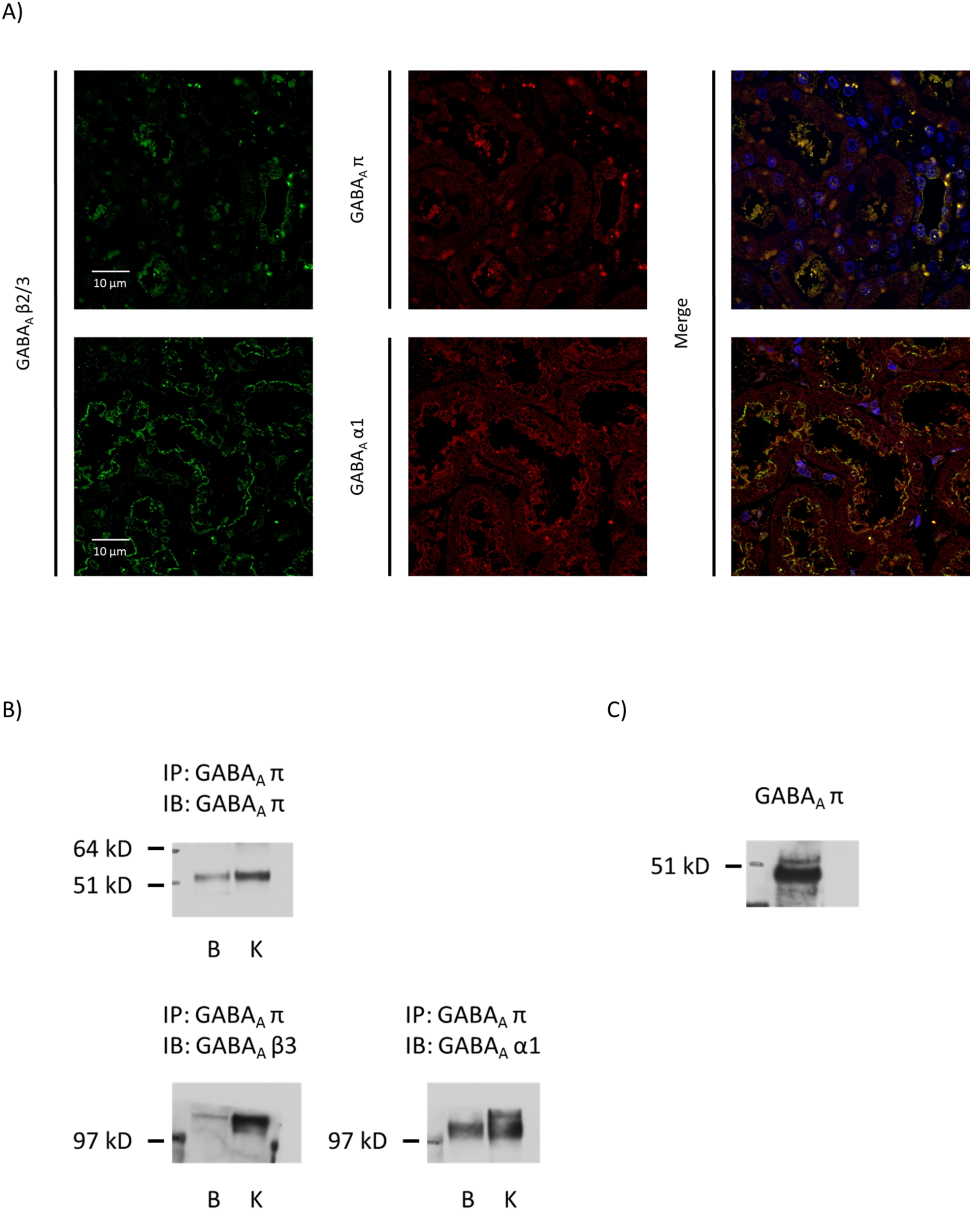
GABA_A_ α1, β3 and π subunits are co-expressed in rat kidney. A) Immunofluorescent images of GABA_A_ α1, β3 and π subunits with DAPI counterstain. B) Immunoblot of rat kidney cortex protein immunoprecipitated with GABA_A_ receptor π subunit antibody. Molecular sizes of α1 and β3 subunits around 110 kDa suggest dimerization. C) Immunoblot of rat kidney cortex membrane protein for GABA_A_ receptor π subunit.

Messenger RNAs of GABA_A_ receptor subunits, α1, β3, δ, ε and π, were detected by RT-PCR in WKY kidney cortex (Figure 1A). All PCR products showed the theoretical molecular weight and their identities were confirmed by sequencing. Clear single bands were detected for all positive controls (brain or universal RNA). However, mRNAs of GABA_A_ receptor subunits, α2, α3, α4, α5, α6, β1, β2, γ1, γ2, γ3 and θ were not detectable in the kidney cortex. Those genes showing clear mRNA signal, namely, α1, β3 and π subunits, were tested for protein expression by immunoblotting. The major band for α1 subunit in the kidney was around 55 kDa, which was the same as a band of the brain sample on the immunoblot. Immunoblotting using an antibody against β2/3 subunit showed a similar pattern in the brain and kidney, with a major band around the theoretical 55 kDa and a secondary band around 40 kDa. The kidney may express similar or greater amounts of β3 subunit than the brain. Immunoblotting for π subunit detected a major band around 50 kDa for both brain and kidney tissue, with a weak secondary band slightly larger than the major band in the brain and smaller bands for kidney samples (Figure 1B). Then, to elucidate the protein localization, immunofluorescence study was performed for α1, β3 and π subunits (Figures 1C and 2A). Alpha 1 subunit distributed widely in the cortical tubules, especially proximal-like tubules. Specific staining of β3 subunit was observed in the apical region of the proximal-like tubules with tall cells and a brush border-like structure and also in the apical region of distal-like tubules. No staining for β3 subunit was detected in the glomeruli. Pi subunit staining was present mostly in the distal-like tubules and less in the proximal-like tubules (Figure 1C).

### GABA_A_ receptor α1, β3 and π subunits co-localized in the apical membranes of cortical tubules

By immunofluorescent observation, co-staining of α1, β3 and π subunits was observed mainly on the apical side of cortical tubules (Figure 2A). Kidney protein immunoprecipitated with π subunit antibody revealed α1 and β3 subunits, and the molecular weights of blotted α1 and β3 subunits were close to 110 kDa, suggesting dimerization (Figure 2B). Pi subunit may be the characteristic GABA_A_ receptor subunit in the kidney, possibly constituting α1/α1/β3/β3/π pentamers. By immunohistochemical observation, it seemed that π subunit may be present in the cytosol. However, because membrane protein preparation of rat kidney clearly showed π subunit signal on the immunoblot (Figure 2C), some of the π subunit molecules are on the membrane and seem to associate with other subunits.

### Both GABA_B_ receptor R1 and R2 subtypes were expressed in rat kidney cortex

**Figure 3:**
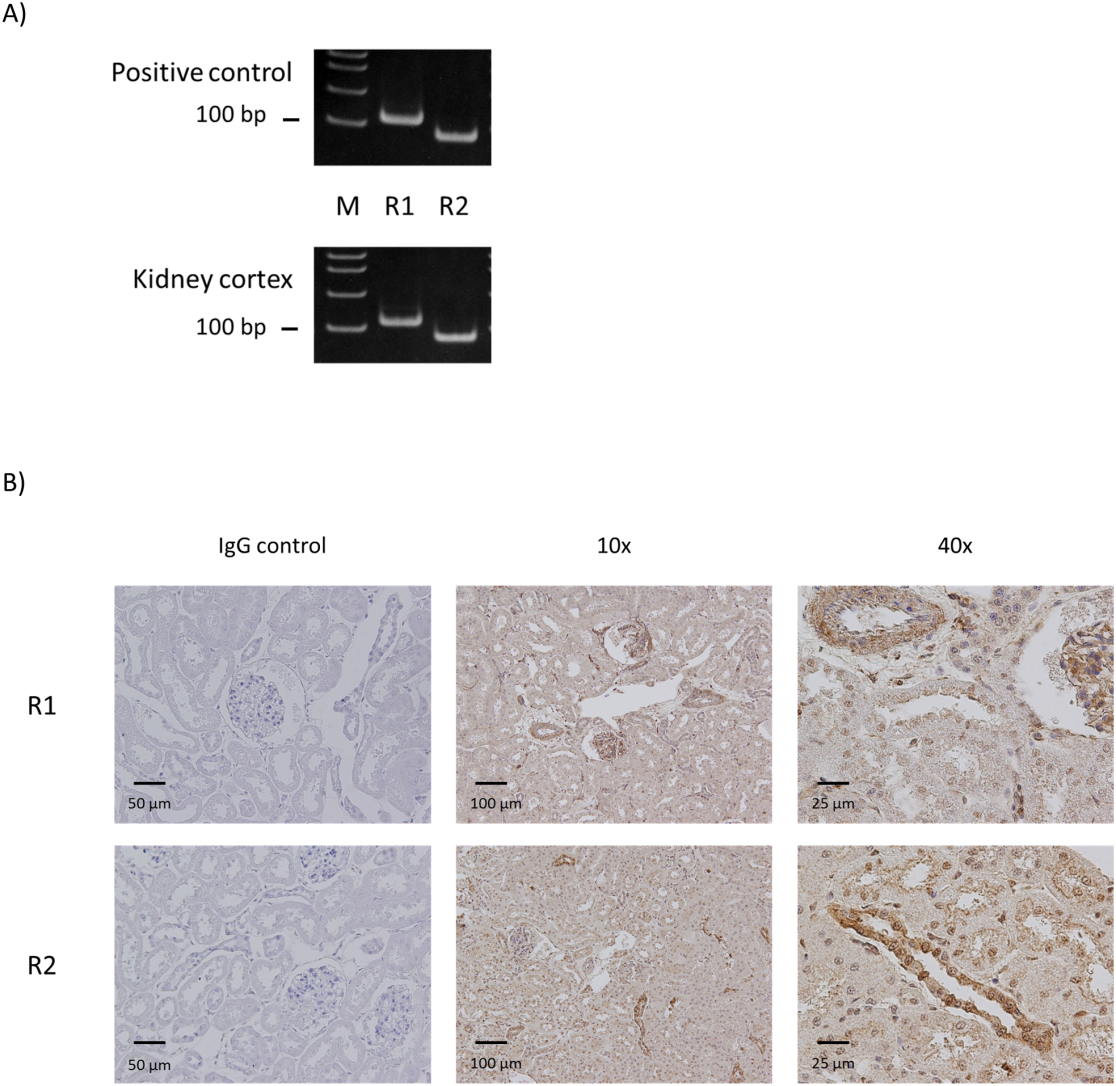
GABA_B_ receptor R1 and R2 subtypes are expressed in rat kidney. A) RT-PCR results for GABA_B_ receptor subtypes in rat kidney cortex. Appropriately-sized bands were detected for GABA_B_ receptor R1 and R2 subtypes in at least five independent experiments. Rat brain RNA or commercial universal RNA was used as positive control. M: molecular marker. B) Immunostaining for GABA_B_ receptor R1 and R2 subtypes in rat kidney cortex. Staining with normal IgG instead of primary antibody served as negative control.

Both types of GABA_B_ receptor, R1 and R2 mRNA, were clearly detected in the kidney cortex (Figure 3A). The primer set used for GABA_B_ receptor R1 mRNA detects all 7 known splice variants of the gene. To visualize the protein localization, immunohistochemistry was performed for both subtypes. In the cortex, R1 subtype was observed in the arteriolae and glomeruli. There was also diffuse signal in most of the tubules, with some stronger signal on the apical side of some of the tubules. GABA_B_ receptor R2 subtype also showed diffuse signal in most of the tubules, with strong signal in the collecting-like tubules, similar to GABA_A_ receptor π subunit, and moderate staining on the apical side of proximal-like tubules (Figure 3B).

### GABA_C_ Receptor ρ1 and ρ2 subunits were detected in rat kidney cortex

**Figure 4:**
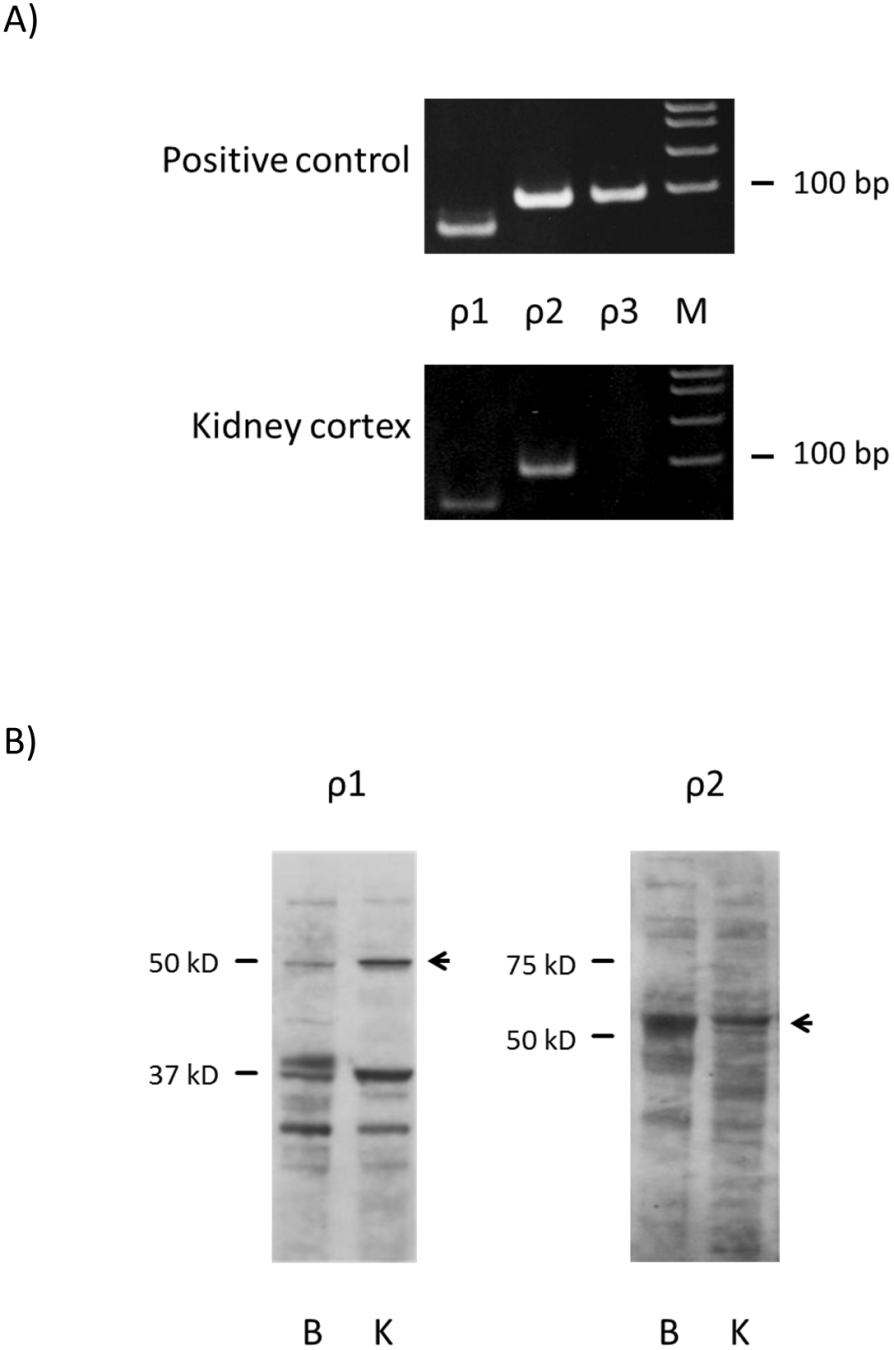
GABA_C_ receptor subunits are expressed in rat kidney. A) RT-PCR results for GABA_C_ receptor subunits in rat kidney cortex. Appropriately-sized bands were detected for GABA_C_ receptor ρ1 and ρ2 subunits in at least five independent experiments. Rat brain RNA or commercial universal RNA was used as positive control. M: molecular marker. B) Immunoblotting for GABA_C_ receptor ρ1 and ρ2 subunits in rat kidney cortex. B: brain control, K: kidney cortex.

Messenger RNAs of GABA_C_ receptor subunits ρ1 and ρ2 were detected by RT-PCR in WKY kidney cortex, but ρ3 was not detectable (Figure 4A). Immunoblotting for ρ1 subunit showed a similar pattern in the brain and kidney, with a major band around the theoretical 48 kDa. The kidney may express similar or greater amounts of ρ1 subunit than the brain. Immunoblotting for ρ2 subunit showed a major band around 50 kDa in both brain and kidney tissues (Figure 4B).

### Two types of GABA-producing enzymes were detected in rat kidney cortex

**Figure 5:**
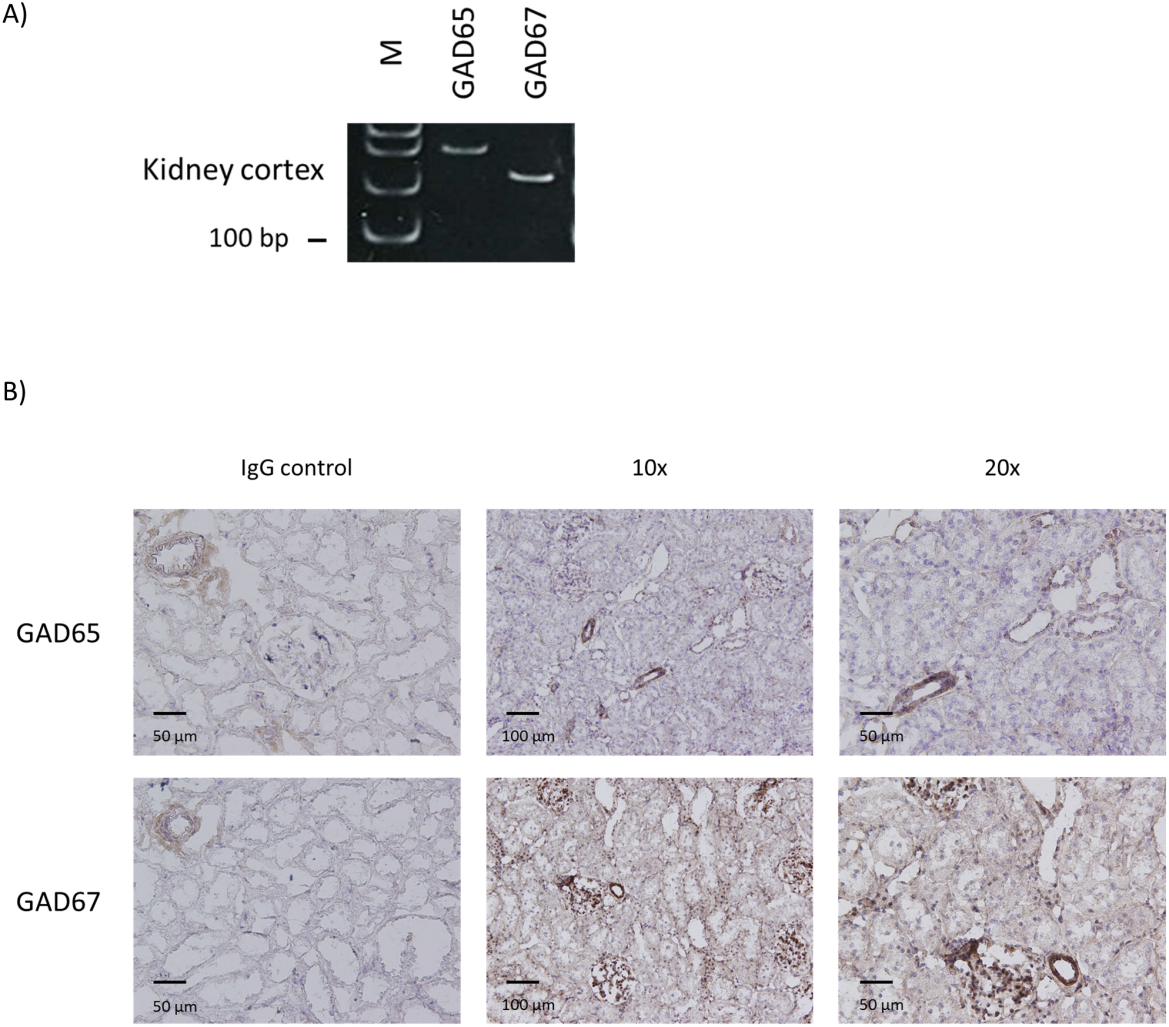
GABA-producing enzymes are expressed in rat kidney. A) RT-PCR results for GAD65 and GAD67 in rat kidney cortex. Appropriately-sized bands were detected for GAD65 and GAD67 in at least five independent experiments. M: molecular marker. B) Immunostaining for GAD65 and GAD67 in rat kidney cortex. Staining with normal IgG instead of primary antibody served as negative control.

Both types of GABA producing enzyme, GAD65 and GAD67 mRNA, were clearly detected in the kidney cortex (Figure 5A). By immunohistochemistry, GAD65 staining was mostly observed in glomeruli and arteriolae. Some weak staining of cortical tubules and stronger staining of vessels and glomeruli were observed for GAD67 (Figure 5B).

### One type of GABA transporter, GAT2, was expressed in rat kidney cortex

**Figure 6:**
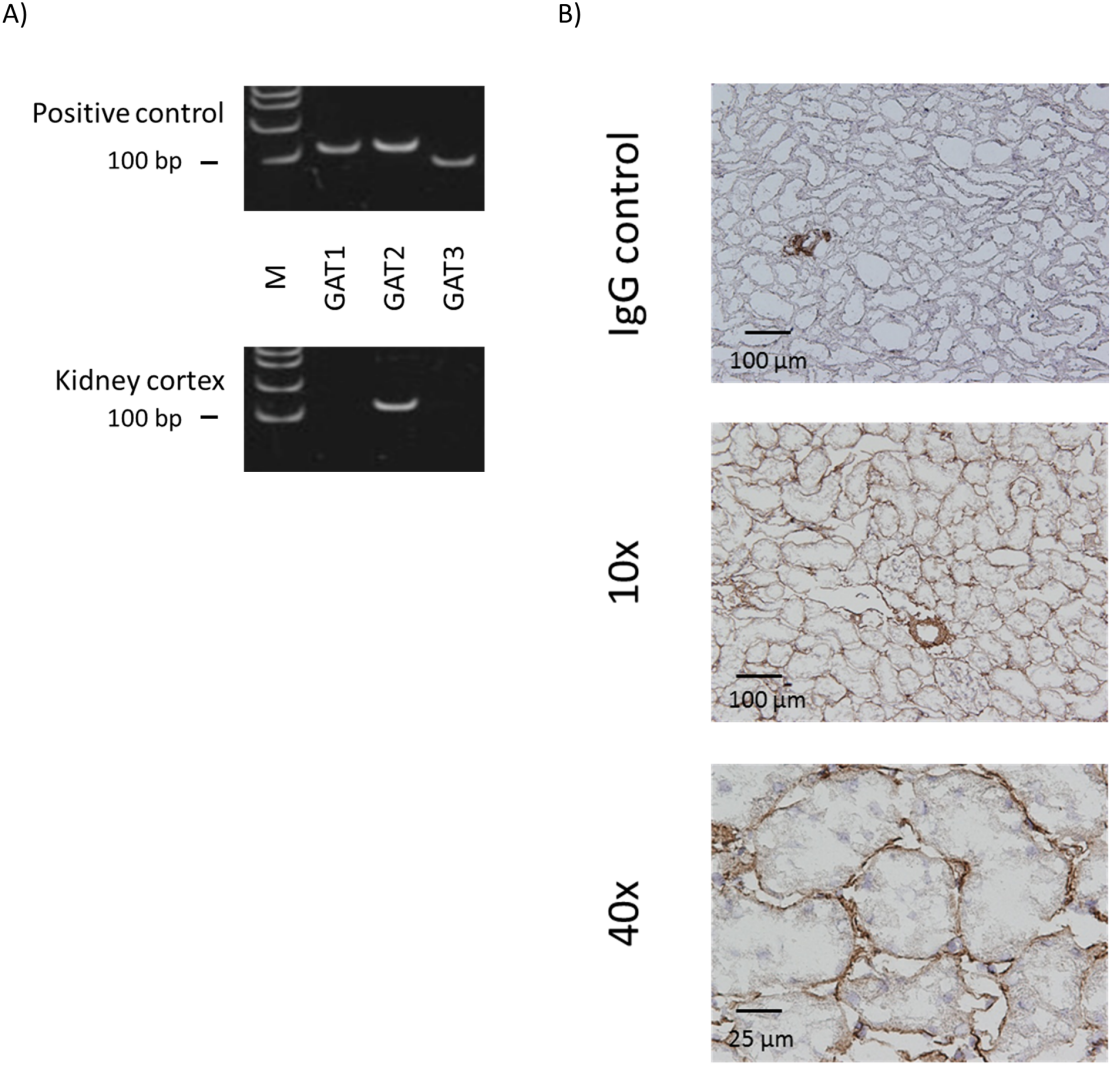
GABA transporters are expressed in rat kidney. A) RT-PCR results for GAT1, GAT2 and GAT3 in rat kidney cortex. Appropriately-sized bands were detected for GAT2 in at least five independent experiments. Rat brain RNA or commercial universal RNA was used as positive control. M: molecular marker. B) Immunostaining for GAT2 in rat kidney cortex. Staining with normal IgG instead of primary antibody served as negative control.

Messenger RNAs of peripheral-type GABA transporter GAT2, were detected, but not of GAT1 and GAT3 (Figure 6A). To confirm the protein localization, immunohistochemistry was performed for GAT2. Specific staining was observed on the basolateral side of cortical renal tubules (Figure 6B).

### GABA degrading enzyme was detected in kidney cortex

**Figure 7:**
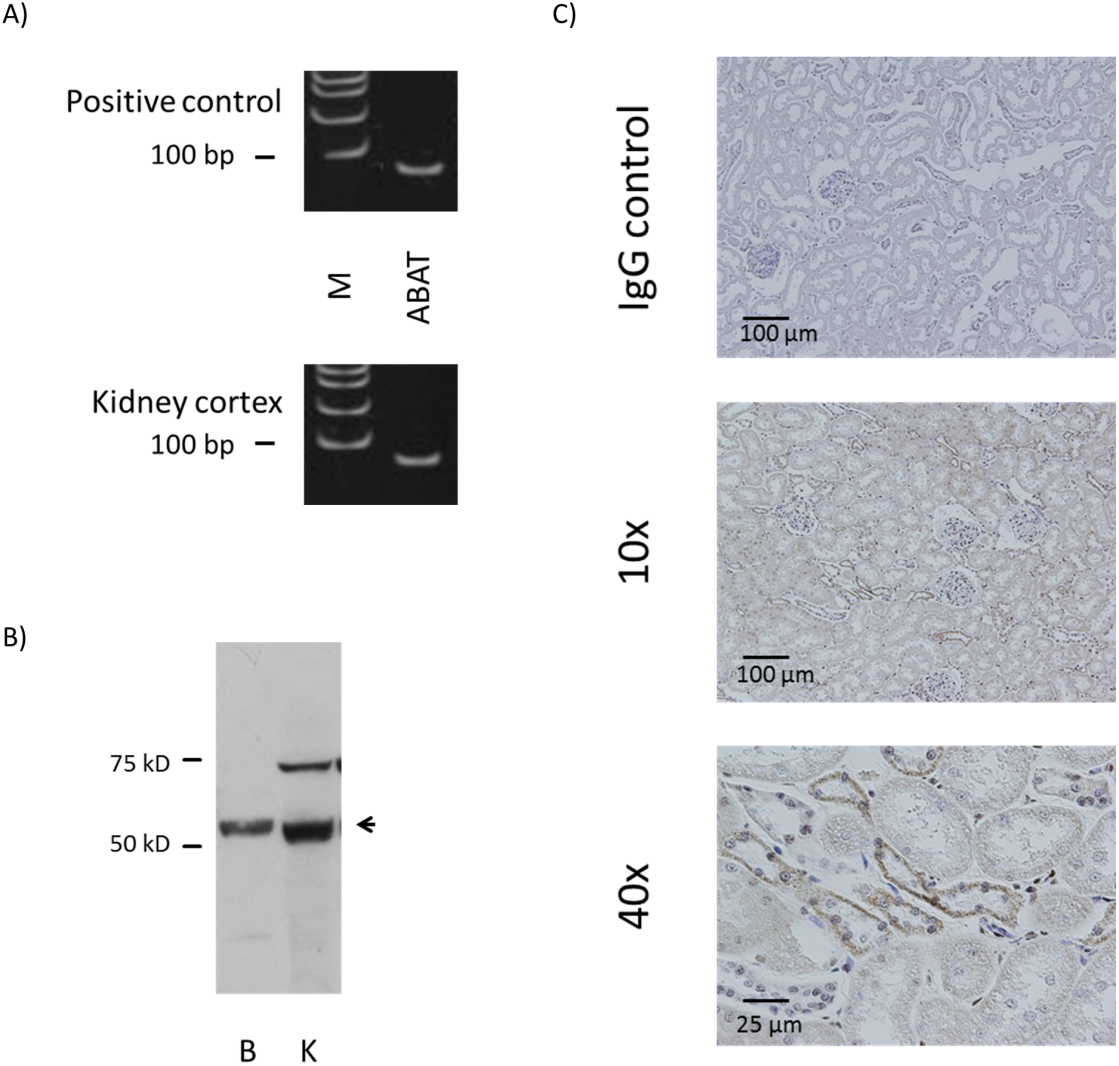
GABA degrading enzyme is expressed in rat kidney. A) RT-PCR results for ABAT in rat kidney cortex. Appropriately-sized bands were detected for ABAT in at least five independent experiments. Rat brain RNA or commercial universal RNA was used as positive control. M: molecular marker. B) Immunoblotting for ABAT in rat kidney cortex. B: brain control, K: kidney cortex. C) Immunostaining for ABAT in rat kidney cortex. Staining with normal IgG instead of primary antibody served as negative control.

Messenger RNA of GABA degrading enzyme ABAT was detected by RT-PCR (Figure 7A) in the kidney cortex using the primer set that amplifies all three known splice variants of the gene. Immunoblotting confirmed the protein expression of ABAT with the major band around 50 kDa for both brain and kidney, and a secondary band larger than the major band in the kidney (Figure 7B). Immunohistochemistry revealed that ABAT staining pattern was also similar to those of GABA_A_ π subunit, present mostly in the distal-like tubules (Figure 7C).

### Renal GABA_A_ receptor expression in hypertensive model rats with salt loading

**Figure 8:**
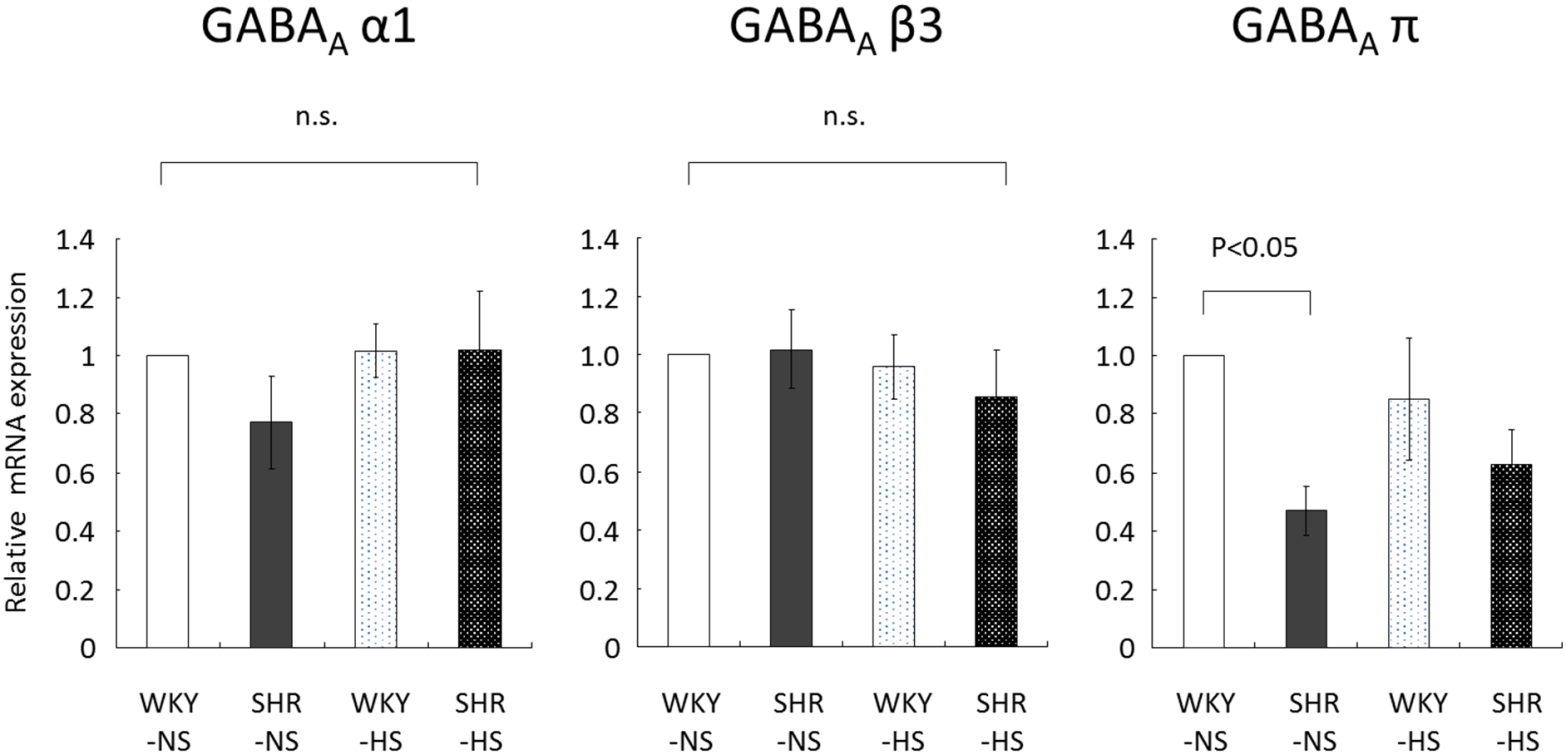
Quantitative RT-PCR results for GABA_A_ receptor subunits in rat kidney cortex. WKY: Wistar-Kyoto rats, SHR: spontaneously hypertensive rats, NS: normal-salt diet, HS: high-salt diet, n.s.: not significant. Statistical significance was assumed at P<0.05.

To explore the possibility of GABA receptor alterations in hypertensive and/or salt-sensitive phenotype, we performed quantitative mRNA analysis and western blots of GABA_A_ subunits using the kidney cortex of WKY and SHR on normal- and high-salt diet. GABA_A_ receptor α1 and β3 subunit expressions in the kidney were not affected by stain or salt loading (Figure 8). In contrast, renal GABA_A_ π subunit (Figure 8) mRNA expression was significantly lower in SHR than in WKY on normal salt diet. The level of GABA_A_ π subunit mRNA in SHR kidney was about half of that in WKY kidney. On high-salt diet, GABA_A_ π subunit mRNA expression tended to be lower in the SHR kidney compared to WKY kidney, but the difference was not significant. Salt loading did not significantly alter renal GABA_A_ π subunit mRNA expression in WKY and SHR. We also performed western blot analysis for GABA_A_ π subunit (WKY normal salt 100±12%, SHR normal salt 121±20%, WKY high salt 100±8%, SHR high salt 95±7%, relative expression normalized to actin, n = 8 rats/group). In contrast to the mRNA difference in WKY and SHR kidneys for GABA_A_ π subunit, we found no significant difference in the expression level GABA_A_ π subunit in the kidney cortex of four groups of rats, WKY and SHR with and without salt loading on the protein level.

### Expressions of GABA-related molecules in human kidney

Next, we examined species differences on the expression of GABA-related molecules in the kidney using human kidney and brain RNA samples. All of the GABA-related molecules consistently detected in rat kidney, specifically, GABA_A_ α1, β3 and π subunits, GABA_B_ R1/R2 subtypes, GABA_C_ subunits ρ1 and ρ2, GAD65 and 67, GAT2, and ABAT were also found in human kidney (data not shown).

## Discussion

In this study, gene expressions of GABA-related molecules were examined in rat kidney cortex. Expressions of GABA_A_ α1, β3, π receptor subunits, GABA_B_ receptor R1 and R2 subtypes, GABA_C_ ρ1 and ρ2 receptor subunits were found at both mRNA and protein levels. Enzymes involved in GABA synthesis, GAD67 and GAD65, GABA transporter, GAT2, and GABA metabolizing enzyme, ABAT, were also expressed in rat kidney.

Our results for GABA_A_ receptor subunits differ in some extent to those previously reported by Sarang et al. They found the expressions of α1, α5, β1, β2, β3, γ1, and γ2 subunits but not of α2, α4, or δ subunits in the kidney cortex from Sprague-Dawley rats [20]. In addition, they found the expression of β2 and β3 subunits in microdissected rabbit proximal tubule S2 segment. We found the expression of α1 and β3 subunits similar to Sarang, et al., but we also found the renal expression of π subunit, which was not examined in their reports. Negative expression results for α5, β1, β2, γ1, and γ2 in our study may be due to either the difference in the detection methods, as Sarang et al used nested PCR whereas our method utilized single amplification, or the difference in rat strain, as we used WKY instead of Sprague-Dawley.

Our report is the first to show a concrete expression of GABA_A_ receptor π subunit in rat and human kidney. To explore the differential expression, Nephromine, a web-based analysis engine freely accessible to academic users at http://www.nephromine.org, was used to study molecular expression in human renal diseases. A search for differential expression of GABA_A_ pi subunit mRNA in human kidney using Nephromine revealed that it may be overexpressed approximately 2 fold in kidneys with diabetic nephropathy (n = 10) compared to those from healthy donors (n = 12). This difference was seen both in tubulointerstitium (fold change: 2.003, P-value: 4.99E-4) and glomeruli (fold change: 2.392, P-value: 0.008, data source [21]). However, GABA_A_ pi subunit expression in hypertension data set did now show a significant difference between normal tissues from tumor nephrectomy (n = 4) and those with nephrosclerosis (n = 14, data source [22]).

We also found the expression of GABA_B_ receptor R1 and R2 subtypes and GABA_C_ receptor ρ1 and ρ2 subunits. As far as we know, this is the first report of GABA_B_ and GABA_C_ receptor protein expressions in the kidney.

It has been shown that GABA_A_ receptor agonist muscimol and GABA_B_ agonist baclofen increased fractional excretion of water and sodium in isolated rat kidney perfusion [16]. Our results suggest the possibility of a novel combination of GABA_A_ receptor subunits, α1, β3 and π, in the kidney, and this combination may be relatively kidney-specific. Muscimol is a potent agonist of GABA_C_ in addition to GABA_A_ receptor [23]. When searching for kidney-specific modulation methods of GABA_A_ receptor, it may be possible in the future to screen for substances that show specific agonistic activity on GABA_A_ receptors with π subunits. Such findings may promote the development of tubule-specific natriuretic agent with less effect on the nervous system. Meanwhile, the function of renal GABA_B_ receptor is not known. As GABA_B_ receptor R1 subtype localizes to glomeruli and arterioles as well as tubules, it may control intra-glomerular perfusion pressure. Also, R2 subtype observed in the distal-like tubules may influence the effects of aldosterone or vasopressin as their co-expression in the principal cells is likely. It has been reported that intrarenal administration of baclofen, a GABA_B_ receptor agonist, has renoprotective effect against ischemia reperfusion injury through the attenuation of renal sympathetic nerve activity [24]. Since overactivation of renal sympathetic nerve activity is associated with tissue renin-angiotensin-aldosterone system overactivity, GABA_B_ activation in renal tubule may alter this phenomenon. Also, the activation of postsynaptic GABA_B_ receptors enhances GABA_A_ currents [25]. Chloride handing in distal tubule and collecting duct modulated by GABA_A_ Cl^−^ channel may be associated with sodium chloride cotransporter and/or epithelial sodium channel. Further study is required to depict a better picture and the precise mechanism of action on GABA receptors expressed in the kidney.

In addition to the GABA receptors, GABA-related enzymes and transporters were also examined in this study. The expression and activity of GAD in the kidney have been reported in the past. Liu et al. found GAD antigen in mouse proximal and distal tubules but not in glomeruli [26]. However, previous reports did not examine GAD subtypes separately. In this study, we found both GAD67 and GAD65 in the kidney tissue using antibodies specific for each subtype.

For GABA transporters, a high level of human GAT2 was found in the kidney and low levels in the brain and lung [27]. Rat GAT2 has been found not only in the brain and retina, but also in the liver, kidney, and heart [28]. GAT1 and GAT3 were not detected in rat kidney, similar to our results [28]. ABAT expression has been demonstrated in rat kidney, and the molecular mass of kidney ABAT was similar to that of liver ABAT and smaller than that of brain ABAT [29].

We have detected renal expressions of major GABA-related molecules, namely receptors, transporters, and synthesizing and metabolizing enzymes. All three GABA_A_ subunits examined in this study, α1, β3, and π, showed staining in the renal tubules. Although the staining patterns are not identical, the presence of all three subunits in the same segment is probable, and this suggests that there may be functional receptor composed of α1β3π subunits, as GABA_A_ receptors formed by α5β3π combination has shown to be functional in transfection experiments [1]. We also found expressions of GAD67 and GAD65 in the renal tubules, which suggest the production of GABA in the kidney. Dopamine in the kidney is a locally-functional, autocrine/paracrine factor known to play a role in the pathophysiology of hypertension [30], [31]. Proximal convoluting tubules produce dopamine which acts on mainly D1-like receptors in the proximal convoluting tubules to induce natriuresis. Renal dopamine synthesis and release is increased by dietary sodium intake and elevation in intracellular sodium concentration, and locally-secreted dopamine in the kidney is responsible for over 50% of sodium excretion during the state of sodium surfeit. Since the current study revealed that rat renal cortex expresses major molecules to account for the entire life cycle of GABA, locally-secreted GABA, similar to dopamine, may act in an autocrine/paracrine fashion in the kidney to induce natriuresis and to lower blood pressure.

In conclusion, rat kidney was found to harbor all major GABA-related components. This suggests the possible existence of a local GABAergic system with an intra-renal GABA production, secretion and function in an autocrine/paracrine manner. Further study is necessary to elucidate the precise localization and function of these renal GABA system molecules. As GABA has been suggested to elicit natriuresis and to lower blood pressure, the research on GABA-related molecules in the kidney is promising as a source of new important information on the pathophysiology of hypertension.
